# Percutaneous endovascular arteriovenous fistula: A systematic review and meta-analysis

**DOI:** 10.3389/fcvm.2022.978285

**Published:** 2022-09-06

**Authors:** Ji-Bo Sun, Chun-Cheng Liu, Xi Shen, Qin Chen, Cheng-Liang Xu, Tian-Lei Cui

**Affiliations:** ^1^Department of Nephrology, West China Hospital/West China School of Medicine, Sichuan University, Chengdu, China; ^2^Department of Ultrasound, West China Hospital, Sichuan University, Chengdu, China; ^3^Department of Endocrinology and Nephrology, 363 Hospital, Chengdu, China; ^4^Department of Nephrology, Wuwei People’s Hospital, Wuwei, China

**Keywords:** percutaneous, endovascular, surgical, arteriovenous fistula, meta-analysis

## Abstract

**Objective:**

Currently, percutaneous endovascular creation of arteriovenous fistula (AVF) shows excellent outcomes. However, few systematic research evidence to support clinical decision making on the benefit of endovascular AVF is available. The purpose of this study was to evaluate the efficacy and safety of endovascular AVF (endoAVF) in patients with renal failure.

**Methods:**

We searched the Medline, Embase, Cochrane Library, and ClinicalTrials.gov databases for studies on endovascular or endovascular versus surgery for the creation of AVF. Two reviewers independently selected studies and extracted data. A systematic review and meta-analysis were performed by Review Manager 5.4 software (Revman, The Cochrane Collaboration, Oxford, United Kingdom) and Stata 15.0 (Stata Corp, College Station, TX, United States).

**Results:**

A total of 14 case series and 5 cohort studies, with 1,929 patients, were included in this study. The technique success was 98.00% for endoAVF (95% CI, 0.97–0.99; *I*^2^ = 16.25%). There was no statistically significant difference in 3 cohort studies between endovascular and surgical AVF for procedural success (OR = 0.69; 95% CI, 0.04–11.98; *P* = 0.80; *I*^2^ = 53%). The maturation rates of endoAVF were 87.00% (95% CI, 0.79–0.93; *I*^2^ = 83.96%), and no significant difference was observed in 3 cohort studies between the 2 groups (OR = 0.73; 95% CI, 0.20–2.63; *P* = 0.63; *I*^2^ = 88%). Procedure-related complications for endoAVF was 7% (95% CI, 0.04–0.17; *I*^2^ = 78.31%), and it did not show significant difference in 4 cohort studies between the 2 groups (OR = 1.85; 95% CI, 0.37–9.16; *P* = 0.45; *I*^2^ = 59%).

**Conclusion:**

The endovascular creation of AVF is potentially effective and safe. These important data may provide evidence to support clinicians and patients in making decisions with endovascular AVF. But further research is great necessary due to lack of randomized controlled studies.

## Introduction

Hemodialysis is the main treatment for patients with end-stage renal disease and successful creation of vascular access is of necessity. Currently, three vascular accesses are commonly used, including AVF, arteriovenous grafts (AVGs), and central venous catheters (CVCs) (1). Given the low risk of infection, high maturation, and low thrombosis, AVF is the preferred vascular access for hemodialysis (2). Traditionally, surgery is performed to establish AVF, but surgical AVF (sAVF) is still at risk of maturation failure and low patency (3, 4). Multiple interventions are imperative to promote maturation and patency of fistulae (5, 6). These factors result in increasing medical burden and reducing lifespan of patients.

In recent years, percutaneous endovascular techniques, WavelinQ (Becton Dickinson, New Jersey) and Ellipsys (Avenu Medical, California), have been used to create AVF with minimal invasiveness (7). The devices allowed more clinicians to perform AVF independently due to the simple operation, which potentially reduced the waiting time and allowed patients to avoid surgical procedures. Moreover, endoAVF creation has shown very promising results in several studies, with high rates of technique success, maturation, and few procedure-related complications (8–26). However, a comprehensive and systematic research evidence of endoAVF is still lacking. Therefore, we conducted a meta-analysis to systematically review the benefits of percutaneous endoAVF creation in patients with end-stage renal disease.

## Methods

This study protocol was conducted strictly in accordance with the preferred reporting items of the systematic review and meta-analysis guidelines (27) (Supplementary Table 1).

### Eligibility criteria

The studies included randomized or non-randomized controlled studies involving the effectiveness and safety of percutaneous endoAVF creation. The included studies with clinical outcomes such as technical success rates, maturation rates, patency rates, or procedure-related complications. We excluded literature reviews, letters, expert opinions, editorials, case series with fewer than 10 patients, articles without complete data, animal and laboratory studies. The studies with only surgical creation of AVF are also excluded.

### Search strategy

A systematic and comprehensive search of the PubMed, Embase, Cochrane Library, and ClinicalTrials.gov databases was conducted by two reviewers from the date of inception of the database to April 20, 2022, to identify all relevant published articles. Studies published in non-English languages were excluded. The following keywords were used in PubMed: “hemodialysis,” “arteriovenous fistula,” “AVF,” “percutaneous,” and “endovascular.” The search strategy for all databases can be provided in Supplementary Appendix 1.

### Study selection and data extraction

Two of us independently and comprehensively screened the title and abstract of each article, and those identified by the initial screening needed to be verified again in full to clarify that the article met the inclusion criteria. If any disagreement was encountered, consensus was reached by discussion among all researchers. Two reviewers independently extracted data from the text, tables and images of the included studies. For each study, we collected first author, year of publication, study design, surgical device, surgery time, characteristics of patients (including sex, age, BMI, diabetes, hypertension), technique success rates, maturation rates, primary and secondary patency at 6 and 12 months, procedure-related complications, and reintervention rates.

### Endpoints and definitions

The primary endpoints were procedure and maturation rates. The secondary endpoints included patency, reintervention, and related-operation complications. Technique success was defined as good anastomosis of the vein and artery and no procedure-related complications. Maturation was defined as ultrasonographic findings of minimum access vessel internal diameter ≥ 5 mm and AVF blood flow ≥ 500 ml/min or successful dialysis using two-needle (28). Patency rates were defined as in Sidawy et al. (29). The reinterventions were to promote the maturation of AVF or maintain the patency of vascular access. The operation-related complications were defined as any unintended medical event directly caused by the operation or device. The main categories included bleeding, pseudoaneurysm, thrombosis, arterial dissection, infection, hematoma, and steal syndrome.

### Quality assessment of included studies

Two reviewers independently assessed the quality of each study. The Newcastle-Ottawa measure was used to evaluate the cohort study, which involved three main components: study cohort selection, cohort comparability, and outcomes ascertainment, with a maximum score of 9 (30). For case series we used an 18-item tool (Supplementary Table 2) with a modified Delphi technique for quality assessment (31). The results of the quality evaluation are presented in Supplementary Tables 3, 4.

### Statistical analyses

The meta-analysis of case series was performed by Stata 15.0, while cohort studies were conducted with Review Manager 5.4. Heterogeneity across studies was evaluated by the *I*^2^ statistic. *I*^2^ values ranged from 0 to 100%; 25 to 50% of *I*^2^ values were considered to have moderate heterogeneity, and *I*^2^ > 75% showed high heterogeneity (32). Considering the characteristics of case series studies, we selected a random effects model for the pooling of results. We also used funnel plots to assess publication bias of the main outcomes. *P* < 0.05 was considered to be statistically significant.

## Results

**FIGURE 1:**
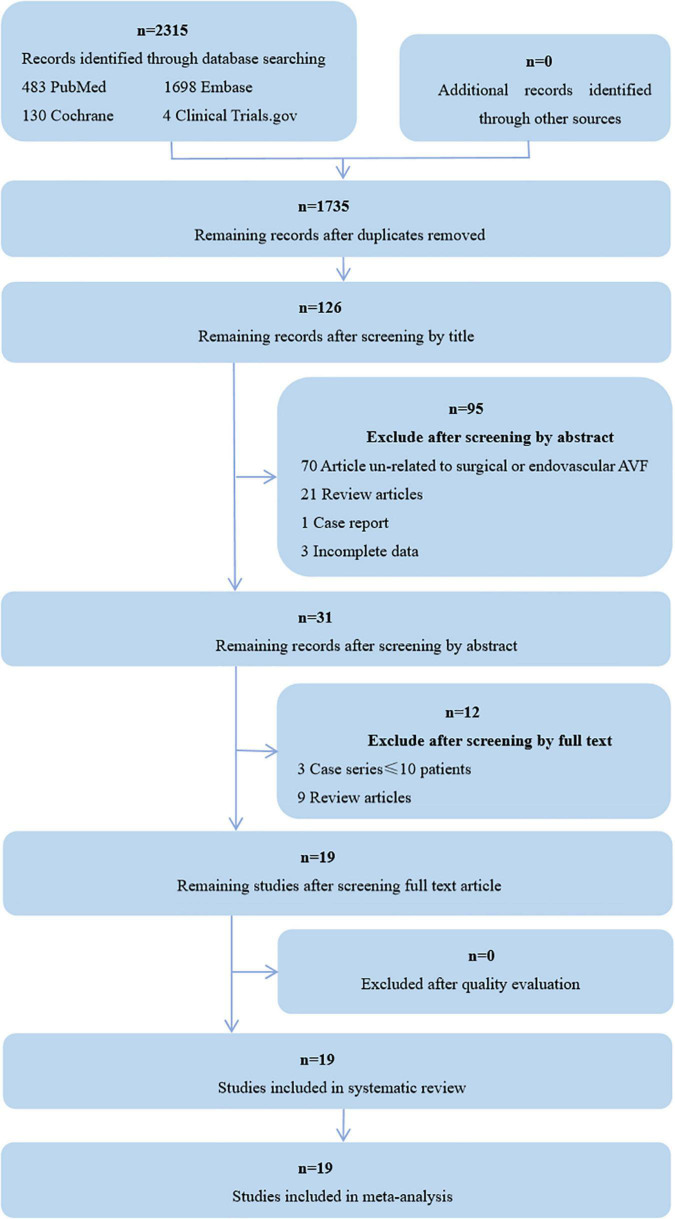
Flow diagram shows studies selection. AVF, arteriovenous fistula.

Initially, 2,315 papers were found by the search strategy, 580 articles were excluded after removing duplications, 1,704 were excluded by title, abstract, paper type, and incomplete data, 12 were excluded by screening by full text, and 19 were finally included for a systematic review and meta-analysis (Figure 1). Among them, 14 were case series, 4 were retrospective cohort studies, and one was prospective cohort study. The detailed characteristics of the included studies are presented in Table 1.

**TABLE 1:** Characteristics of included studies.

Author, year	Design	Patients (p/s)	Device (p/s)	PT, min (p/s)	Age (p/s)	Male, *n* (p/s)	BMI, kg/m^2^	DM, *n* (p/s)	HTN, *n* (p/s)
Mordhorst et al. (26)	RC	61/308	WavelinQ/S	55.0**^a^**/NR	64.0^a^/64.2	46/196	NR	36/186	50/286
Harika et al. (23)	RC	107/107	Ellipsys/S	NR	63.6 ± 15.41/63.5 ± 15.69**^b^**	66/65	27.2 ± 5.78/26.8 ± 5.95**^b^**	66/52	99/102
Osofsky et al. (25)	RC	24/62	Ellipsys/S	60 ± 40/56 ± 25 **^b^**	56.7 ± 22.6/62.5 ± 13.2**^b^**	12/32	28.8 ± 6.8/30.5 ± 6.7**^b^**	18/46	21/58
Shahverdyan et al. (24)	RC	89/69	Ellipsys/S	14.0/74.0**^a^**	66.0/67.9**^a^**	58/35	26.2/28.7**^a^**	32/33	NR
Inston et al. (22)	PC	30/40	WavelinQ/S	NR	57 ± 15/54 ± 17**^b^**	25/29	NR	NR	NR
Berland et al. (21)	C	120/NR	WavelinQ/NR	NR	54.6 ± 15.9**^b^**/NR	97/NR	27.0 ± 6.6**^b^**/NR	NR	NR
Kitrou et al. (20)	C	30/NR	WavelinQ/NR	NR	55.3 ± 13.6**^b^**/NR	30/NR	NR	15/NR	21/NR
Zemela et al. (19)	C	32/NR	WavelinQ/NR	120.0**^a^**/NR	60.2**^ a^**/NR	23/NR	32.5**^a^**/NR	21/NR	31/NR
Shahverdyan et al. (17)	C	100/NR	Ellipsys+ WavelinQ/NR	18.0**^a^**/NR	64.18 ± 14.18**^b^**/NR	69/NR	27.21 ± 6.70**^b^**/NR	37/NR	NR
Hull et al. (15)	C	62/NR	Ellipsys/NR	NR	64 ± 14**^b^**/NR	34/NR	30.7 ± 9.0**^b^**/NR	55/NR	57/NR
Beathard et al. (18)	C	105/NR	Ellipsys/NR	NR	56.2**^a^**/NR	77/NR	31.21**^a^**/NR	NR	NR
Mallios et al. (16)	C	232/NR	Ellipsys/NR	15.0**^a^**/NR	64**^a^**/NR	148/NR	NR	140/NR	NR
Berland et al. (14)	C	32/NR	WavelinQ/NR	NR	51 ± 13**^b^**/NR	31/NR	NR	17/NR	27/NR
HeBiBi et al. (13)	C	34/NR	Ellipsys/NR	NR	60.6**^a^**/NR	20/NR	NR	12/NR	33/NR
Mallios et al. (12)	C	34/NR	Ellipsys/NR	NR	64.0**^a^**/NR	22/NR	NR	21/NR	NR
Hull et al. (11)	C	107/NR	Ellipsys/NR	23.7 ± 11.3**^b^**/ NR	56.7 ± 12.0**^b^**/ NR	78/NR	31.18 ± 7.13**^b^**/ NR	69/NR	105/NR
Lok et al. (9)	C	80/NR	WavelinQ/NR	NR	60.1 ± 13.1**^b^**/NR	54/NR	28.1 ± 6.1**^b^**/NR	49/NR	73/NR
Hull et al. (10)	C	26/NR	Ellipsys/NR	18.4**^a^**/NR	45.5 ± 13.6**^b^**/NR	10/NR	26.7 ± 5.1**^b^**/NR	17/NR	24/NR
Rajan et al. (8)	C	33/NR	WavelinQ/NR	NR	51.0 ± 11.4**^b^**/NR	20/NR	24.3 ± 3.8**^b^**/NR	19/NR	NR
**AV anastomosis (p/s)**			**Preoperative requirement (p/s)**			**Intraoperative/Postoperative adjunctive techniques (p/s)**			

P: Ulnar artery-vein/radial artery-vein/interosseous artery-vein S: Radiocephalic/brachiocephalic			P: Radial/ulnar/brachial vein > 2 mm S: Radial artery ≥ 1.5 mm, cephalic vein ≥ 2.0–2.5 mm			NR			
P: PRA and perforating vein of the elbow S: Radiocephalic/brachiocephalic			Vein and/or artery > 2 mm			NR			
P: Radiocephalic S: Brachiocephalic			P: PRA ≥ 2 mm, DCV ≥ 2 mm, PRA to DCV proximity of ≤ 1.5 mm S: Cephalic vein ≥ 2.5 mm, brachial artery ≥ 3.0 mm			P: Coil embolization, ligation, superficialization or transposition S: NR			
P: Radial-cephalic/basilic veins S: Radial/ulnar/brachial-cephalic/ basilic vein			Vein and artery ≥ 2 mm, a distance of ≤ 1.5 mm between PRA and DCV			P: Balloon-angioplasty of the anastomosis S: NR			
P: Ulnar artery-vein, interosseous artery-vein S: Radiocephalic			Vein and artery ≥ 2 mm			P: Angioplasty, stent, soiling and transposition S: Angioplasty, stent			
Ulnar artery-vein or concomitant radial-vein			Artery and vein ≥ 2 mm			Superficialization, coiling, stenting			
Ulnar artery-vein or radial artery-vein			Artery and vein ≥ 2.0 mm, superficial outflow vein ≥ 2.5 mm			Coiling, angioplasty, coiling + PTA, stenting			
Ulnar artery-vein or radial artery-vein			Inflow artery > 2 mm, outflow superficial vein > 2.5 mm, a deep vein > 2 mm			Surgical and/or endovascular intervention			
Radial/ulnar artery-cephalic, basilic, cephalic and brachial vein			Brachial artery > 2 mm, radial or ulnar artery > 2 mm			Coil embolization, balloon dilation			
PRA-Cubital and brachial vein			Vein/artery ≥ 2.0 mm			Balloon dilation, stenting, embolization,			
PRA-vein			NR			Balloon dilation			
PRA-perforating vein			PRA > 2 mm, PVE > 2.0 mm and a distance between these vessels < 1.5 mm			Balloon angioplasty, superficializations			
Ulnar artery-vein or radial artery-vein			Target vein/artery ≥ 2.0 mm			NR			
PRA-DCV			Distance between DCV and PRA < 1.5 mm, DCV and PRA ≥ 2 mm			Superficialization			
PRA-perforating vein			Radial artery > 2.0 mm, perforating vein > 3.0 mm			Superficialization			
Upper-extremity AVF			Target vein/artery ≥ 2.0 mm			Balloon dilation, embolization, ligation, transposition			
Ulnar artery-vein			Target vein/artery ≥ 2.0 mm			Coil embolization			
PRA-perforating vein			Radial artery > 2.0 mm, adjacent vein > 2.0 mm			Coil embolization			
Ulnar artery-vein			Target vein/artery ≥ 2.0 mm			NR			

PT, procedure time; BMI, body mass index; DM, diabetes mellitus; HTN, hypertension; P, percutaneous; S, surgery; RC, retrospective cohort; PC, prospective cohort; C, case series; NR, not reported; ^a^Data are expressed as mean; ^b^Data are expressed as mean standard ± deviation; AV, artery-vein; PRA, proximal radial artery; DCV, deep communicating vein; PTA, percutaneous transluminal angioplasty; PVE, perforating vein of the elbow.

### Primary outcomes

#### Technique success

**FIGURE 2:**
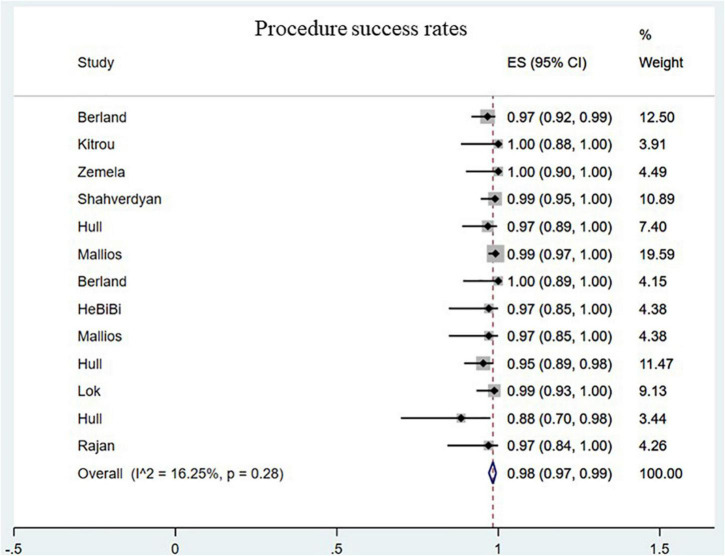
Pooled procedure success rates. CI, confidence interval; ES, effect size.

**FIGURE 3:**
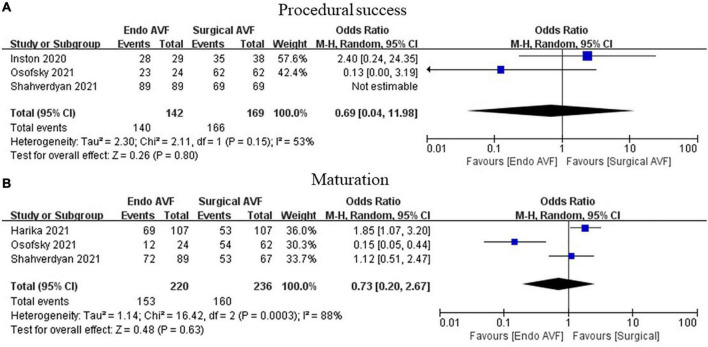
Meta-analysis of procedural success and maturation. **(A)** Above we have marked procedural success and **(B)** marked maturation. AVF, arteriovenous fistula; M-H, Mantel-Haenszel.

The procedure success rate was 98.00% for endoAVF (95% CI, 0.97–0.99; *I*^2^ = 16.25%; Figure 2) (8–17, 19–21). There was no statistically significant difference between endovascular and surgical creation of AVF in the procedural success (OR = 0.69; 95% CI, 0.04, 11.98; *P* = 0.80; *I*^2^ = 53%; Figure 3A) (22, 24, 25).

#### Maturation rates

**FIGURE 4:**
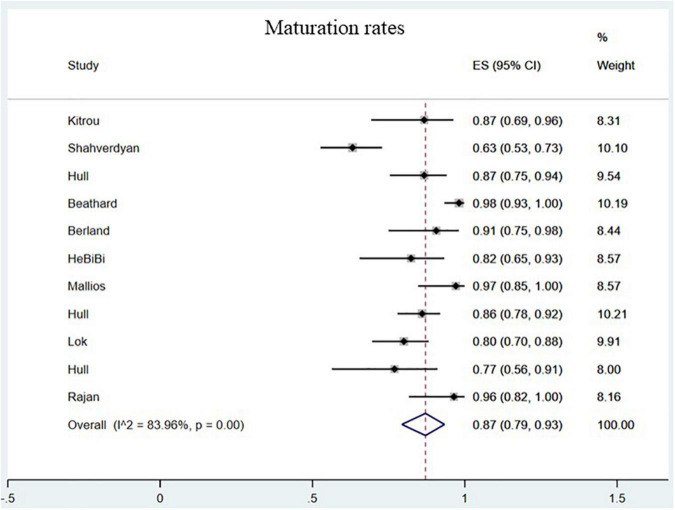
Pooled maturation rates. CI, confidence interval; ES, effect size.

The maturation rates of endovascularly created AVF were 87.00% (95% CI, 0.79–0.93; *I*^2^ = 83.96%; Figure 4) (8–15, 17, 18, 20). No statistically significant difference in maturation was observed between the 2 groups (OR = 0.73; 95% CI, 0.20–2.63; *P* = 0.63; *I*^2^ = 88%; Figure 3B) (23–25).

### Secondary outcomes

#### Operation-related complications

Procedure-related complications for endoAVF showed 7% (95% CI, 0.04–0.17; *I*^2^ = 78.31%; Supplementary Figure 1) (8–17, 19–21), and there was no significant difference between the endovascular and surgical AVF (OR = 1.85; 95% CI, 0.37–9.16; *P* = 0.45; *I*^2^ = 59%; Supplementary Figure 2A) (23–26).

#### Reintervention rates

The reintervention rates for endoAVF were 51.00% (95% CI, 0.30–0.70; *I*^2^ = 95.67%; Supplementary Figure 3) (9–13, 15, 17, 19–21). And there was no significant difference between the 2 groups (OR = 2.42; 95% CI, 0.50–11.83; *P* = 0.27; *I*^2^ = 89%; Supplementary Figure 2B) (22, 24, 25).

#### Patency rates

The 6-month primary patency rates of endoAVF were 38% (95% CI, 0.28–0.48; *I*^2^ = 0.00%; Supplementary Figure 4) (14, 15), and no significant difference was found between the 2 groups (OR = 0.75; 95% CI, 0.20–2.86; *P* = 0.68; *I*^2^ = 81%; Supplementary Figure 5A) (22, 24). The 6-month secondary patency rates of endoAVF were 90% (95% CI, 0.82–0.96; *I*^2^ = 63.34%; Supplementary Figure 6) (8, 10, 14, 15, 18), and no significant difference was observed between the 2 groups (OR = 1.58; 95% CI, 0.80–3.13; *P* = 0.19; *I*^2^ = 0.00%; Supplementary Figure 5B) (22, 24). The 12-month primary patency rates of endoAVF were 62% (95% CI, 0.38–0.83; *I*^2^ = 96.57%; Supplementary Figure 7) (9, 11, 16, 17). There was no statistically significant difference between the endovascular and surgical AVF for the 12-month primary patency (OR = 0.55; 95% CI, 0.22–1.40; *P* = 0.21; *I*^2^ = 79.00%; Supplementary Figure 5C) (22–24). The 12-month secondary patency rates of endoAVF were 87% (95% CI, 0.78–0.94; *I*^2^ = 86.69%; Supplementary Figure 8) (9–11, 16–18, 20), and there was no statistically significant difference between the 2 groups (OR = 1.44; 95% CI, 0.85–2.44; *P* = 0.17; *I*^2^ = 0.00%; Supplementary Figure 5D) (22–24).

## Discussion

In the current study, a comprehensive systematic review and meta-analysis was performed to assess the safety and efficacy of endoAVF. The study has shown percutaneous endovascular creation of AVF with a higher success and maturation rate, few related-procedure complications, and less reinterventions to promote maturation or maintain access patency.

Although AVF is the preferred access in hemodialysis patients, low maturation rate is a challenging problem. Some multiple-center prospective cohort studies have shown AVF maturation success rates of only 60 to 67% (33, 34). And studies have indicated that 35 to 50% of surgical AVF required intervention prior to successful use in hemodialysis (35–37). Therefore, finding new operation methods to improve AVF maturation and reduce interventions is of great significance. Currently, the WavelinQ system and the Ellipsys system, are the two endovascular devices. For the WavelinQ system, a dual catheter plays an important role in creating an anastomosis between the deep artery and vein in the proximal forearm (7). Whilst the Ellipsys system, is a 6-Fr single-catheter access system that provides heat and pressure to anastomosis between the proximal radial artery and the penetrating vein (38). Compared with surgical creation of AVF, endoAVF with minimal trauma showed better outcomes, which may provide a reliable alternative to creation of AVF (39). Furthermore, in a systematic review and meta-analysis on the cost of postoperative maintenance of open versus endovascular AVF creation, fewer post-procedure interventions for endoAVF patients, which directly contributed to significantly lower maintenance costs for these patients in the first year after the creation of endoAVF (40).

In our study, the success and maturation rates of AVF were 98.00 and 87.00%, respectively, which is similar to the results of a previous study. In Yan Wee et al. reported a systematic review and meta-analysis involving seven studies of endoAVF, with a technique success of 97.50% and maturation rate of 89.27% at 90 days, respectively (41). However, given the large number of comprehensive data included in this study, the evidence we supported would be more reliable and scientific in endoAVF for clinicians and patients. The higher success and maturation rates of endoAVF may be related to the less invasive procedure, types of AVF anastomosis and the better vascular conditions. EndoAVF is created by skin direct puncture with a needle and special device, rather than through open surgery, so it is less invasive compared to surgery and avoids excessive manipulation of the blood vessels during open surgery that can lead to vascular damage, which in turn can produce endothelial hyperplasia and lead to stenosis (42). EndoAVF anastomosis is performed using the vein side to arterial side (side-to-side) compared to the commonly used surgical vein end to arterial side (end-to-side), and side-to-side anastomosis approach has been shown to be effective in reducing stenosis of juxta-anastomosis (42, 43). Intimal hyperplasia may be due to turbulent flow at the juxta-anastomosis, combined with lower and oscillating wall shear stress (44), so a larger anastomosis angle may be beneficial for upper arm fistulas (45). The side-to-side anastomosis created in endovascular AVF may reduce wall shear stress, thereby reducing neointimal hyperplasia. In addition, patients with endoAVF are in better vessel quality, which improves the success rate and maturity of the procedure to some extent. And no statistically significant difference was observed between the 2 groups for the success rate and complications, which is similar to the previous results (46). In addition, maturation, reintervention, and patency rates were not significant, but findings may be because of our limited number of studies. In this study, the procedure-related complications and re-intervention rates were low due to a minimally invasive operation. The endoAVF was created percutaneously through needles and special devices rather than surgery, so it lacked traditional surgical scars and may not be easily recognized by health care providers. As a result, blood pressure cuff inflation or intravenous needle placement may inadvertently damage endoAVF outflow veins. Therefore, patient education must be focused on protecting the endoAVF.

There are some limitations in this study. First, the findings still need to be further validated in future due to lack of randomized controlled trials; In subgroup analysis only 5 cohort studies were included, may resulting in non-statistical difference and high heterogeneity and the results may not be solid enough. Second, the maturation rates in this study were not strictly defined as the specific time, and maturation was defined as maturity during the follow-up period, which may make the study results biased. Third, the endoAVF has a higher standard for vessel diameter and is only used between adjacent arteries and veins, which may lead to biased selection of study patients and thus exaggerate the results.

Endovascular AVF could be a feasible and safe approach. These key data may provide evidence for clinicians and patients with renal failure to make decisions regarding endoAVF. However, given the lack of high-quality randomized controlled trials, further evaluations are necessary.

## Data availability statement

The original contributions presented in this study are included in the article/Supplementary material, further inquiries can be directed to the corresponding author.

## Author contributions

T-LC and J-BS: conception and design. T-LC: administrative support and provision of study materials. J-BS, C-CL, and XS: collection and assembly of data. J-BS, XS, QC, and C-LX: data analysis and interpretation. All authors contributed to the manuscript writing and final approval of manuscript.
