# Supplementary material for: Percutaneous endovascular arteriovenous fistula: A systematic review and meta-analysis
Source: Front Cardiovasc Med. 2022 Sep 6;9:978285. doi: 10.3389/fcvm.2022.978285 (PMC9486211; doi:10.3389/fcvm.2022.978285)
Supplement: Supplementary file 1 [file Data_Sheet_1.pdf]

Appendix 1. Detailed search strategies

PubMed <1946 to April 20, 2022>

1 "Renal Dialysis"[Mesh] 121,845

2 (((((((((Dialyses, Renal[Title/Abstract])) OR (Renal Dialyses[Title/Abstract])) OR (Dialysis, Renal[Title/Abstract])) OR (Hemodialysis[Title/Abstract])) OR (Hemodialyses[Title/Abstract])) OR (Dialysis, Extracorporeal[Title/Abstract])) OR (Dialyses, Extracorporeal[Title/Abstract])) OR (Extracorporeal Dialyses[Title/Abstract])) OR (Extracorporeal Dialysis[Title/Abstract]) 116,235

3 1 OR 2 162,248

4 "Arteriovenous Fistula"[Mesh] 15,341

5 (((((Fistula, Arteriovenous[Title/Abstract])) OR (Fistulas, Arteriovenous[Title/Abstract])) OR (Arteriovenous Fistulas[Title/Abstract])) OR (Arteriovenous Aneurysm[Title/Abstract])) OR (Aneurysm, Arteriovenous[Title/Abstract]) 6,662

6 4 OR 5 18,863

7 3 AND 6 2,198

8 "Endovascular Procedures"[Mesh] 138,256

9 (((((((((((((((Endovascular Procedure[Title/Abstract])) OR (Procedure, Endovascular[Title/Abstract])) OR (Procedures, Endovascular[Title/Abstract])) OR (Intravascular Procedures[Title/Abstract])) OR (Intravascular Procedure[Title/Abstract])) OR (Procedure, Intravascular[Title/Abstract])) OR (Procedures, Intravascular[Title/Abstract])) OR (Intravascular Techniques[Title/Abstract])) OR (Intravascular Technique[Title/Abstract])) OR (Technique, Intravascular[Title/Abstract])) OR (Techniques, Intravascular[Title/Abstract])) OR (Endovascular Techniques[Title/Abstract])) OR (Endovascular Technique[Title/Abstract])) OR (Technique, Endovascular[Title/Abstract])) OR (Techniques, Endovascular[Title/Abstract]) 4,955

10 8 OR 9 140,983

11 Percutaneous 172,828

12 10 OR 11 260,159

13 7 AND 11 **483**

Embase <1974 to April 20, 2022>

#1 "hemodialysis'/exp 125783

#2 'dialyses, renal':ab,ti OR 'renal dialyses':ab,ti OR 'dialysis, renal':ab,ti OR 'hemodialysis':ab,ti OR 'hemodialyses':ab,ti OR 'dialysis, extracorporeal':ab,ti OR 'dialyses, extracorporeal':ab,ti OR 'extracorporeal dialyses':ab,ti OR 'extracorporeal dialysis':ab,ti 101873

#3 #1 OR #2 159874

#4 'arteriovenous fistula'/exp 38294

#5 'fistula, arteriovenous':ab,ti OR 'fistulas, arteriovenous':ab,ti OR 'arteriovenous fistulas':ab,ti OR 'arteriovenous aneurysm':ab,ti OR 'aneurysm, arteriovenous':ab,ti 7170

#6 #4 OR #5 40111

#7 endovascular 121512

#8 percutaneous 319557

#9 #7 OR #8 424040

#10 #3 AND #6 AND #9 **1698**

Cochrane Library <--to April 20, 2022>

#1 MeSH descriptor: [Renal Dialysis] explode all trees 5481

#2 (Dialyses, Renal):ti,ab,kw OR (Renal Dialyses):ti,ab,kw OR (Dialysis, Renal):ti,ab,kw OR (Hemodialysis):ti,ab,kw OR (Hemodialyses):ti,ab,kw OR (Dialysis, Extracorporeal):ti,ab,kw OR (Dialyses, Extracorporeal):ti,ab,kw OR (Extracorporeal Dialyses):ti,ab,kw OR (Extracorporeal Dialysis):ti,ab,kw 17221

#3 #1 OR #2 17719

#4 MeSH descriptor: [Arteriovenous Fistula] explode all trees 143

#5 (Fistula, Arteriovenous):ti,ab,kw OR (Fistulas, Arteriovenous):ti,ab,kw OR (Arteriovenous Fistulas):ti,ab,kw OR (Arteriovenous Aneurysm):ti,ab,kw OR (Aneurysm, Arteriovenous):ti,ab,kw 1337

#6 #4 OR #5 1337

#7 (Endovascular):ti,ab,kw (Word variations have been searched) 4210

#8 (Percutaneous):ti,ab,kw (Word variations have been searched) 21799

#9 #7 OR #8 25447

#10 #3 and #6 and #9 **130**

Clinical Trials.gov

Arteriovenous fistula and (percutaneous OR endovascular) **4**

Supplementary Table 1. MOOSE Checklist for Meta-analyses of Observational Studies

Item No	Recommendation	Reported on Page No
Reporting of background should include		
1	Problem definition	4
2	Hypothesis statement	N/A
3	Description of study outcome(s)	4
4	Type of exposure or intervention used	4
5	Type of study designs used	5
6	Study population	5
Reporting of search strategy should include		
7	Qualifications of searchers (eg, librarians and investigators)	5
8	Search strategy, including time period included in the synthesis and key words	5
9	Effort to include all available studies, including contact with authors	5
10	Databases and registries searched	5
11	Search software used, name and version, including special features used (eg, explosion)	Appendix
12	Use of hand searching (eg, reference lists of obtained articles)	N/A
13	List of citations located and those excluded, including justification	Figure 1
14	Method of addressing articles published in languages other than English	N/A
15	Method of handling abstracts and unpublished studies	Figure 1
16	Description of any contact with authors	5
Reporting of methods should include		
17	Description of relevance or appropriateness of studies assembled for assessing the hypothesis to be tested	6
18	Rationale for the selection and coding of data (eg, sound clinical principles or convenience)	6
19	Documentation of how data were classified and coded (eg, multiple raters, blinding and interrater reliability)	5
20	Assessment of confounding (eg, comparability of cases and controls in studies where appropriate)	6
21	Assessment of study quality, including blinding of quality assessors, stratification or regression on possible predictors of study results	6
22	Assessment of heterogeneity	6

23	Description of statistical methods (eg, complete description of fixed or random effects models, justification of whether the chosen models account for predictors of study results, dose-response models, or cumulative meta-analysis) in sufficient detail to be replicated	6
24	Provision of appropriate tables and graphics	Tables and Figures
Reporting of results should include		
25	Graphic summarizing individual study estimates and overall estimate	Figure 1-3
26	Table giving descriptive information for each study included	Table 1 and Table S1
27	Results of sensitivity testing (eg, subgroup analysis)	7-9
28	Indication of statistical uncertainty of findings	7-9

Supplementary Table 2. Major components of the tools for case series assessment

Major components	Judgment
**Study objective**	
1. Is the hypothesis/aim/objective of the study stated clearly in the abstract, introduction, or methods section?	1. Yes, Unclear, No
**Study population**	
2. Are the characteristics of the participants included in the study described?	2. Yes, Partially reported, No
3. Were the cases collected in more than one centre?	3. Yes, Unclear, No
4. Are the eligibility criteria (inclusion and exclusion criteria) for entry into the study explicit and appropriate?	4. Yes, Partially reported, No
5. Were participants recruited consecutively?	5. Yes, Unclear, No
6. Did participants enter the study at a similar point in the disease?	6. Yes, Unclear, No
**Intervention and co-intervention**	
7. Was the intervention clearly described in the study?	7. Yes, Partially reported, No
8. Were additional interventions (co-interventions) clearly reported in the study?	8. Yes, Unclear, No
**Outcome measure**	
9. Are the outcome measures clearly defined in the introduction or methods section?	9. Yes, Partially reported, No
10. Were relevant outcomes appropriately measured with objective and/or subjective methods?	10. Yes, Unclear, No
11. Were outcomes measured before and after intervention?	11. Yes, Unclear, No
**Statistical analysis**	
12. Were the statistical tests used to assess the relevant outcomes appropriate?	12. Yes, Unclear, No
**Results and conclusions**	

13. Was the length of follow-up reported?	13. Yes, Unclear, No
14. Was the loss to follow-up reported?	14. Yes, Unclear, No
15. Does the study provide estimates of the random variability in the data analysis of relevant outcomes?	15. Yes, Unclear or partially reported, No
16. Are adverse events reported?	16. Yes, Partially reported, No
17. Are the conclusions of the study supported by results?	17. Yes, Partially reported, No
**Competing interests and sources of support**	
18. Are both competing interests and sources of support for the study reported?	18. Yes, Partially reported, No

Supplementary Table 3. Results of quality assessment of case series by 18-item tool

Author	Year	1	2	3	4	5	6	7	8	9	10	11	12	13	14	15	16	17	18
Berland	2022	Y	Y	Y	Y	Y	Y	Y	Y	Y	Y	Y	Y	Y	Y	Y	Y	Y	Y
Kitrou	2022	Y	Y	N	Y	Y	Y	Y	Y	Y	Y	Y	Y	Y	Y	N	Y	Y	Y
Zemela	2021	Y	Y	N	N	Y	Y	Y	Y	Y	Y	Y	Y	N	Y	N	Y	Y	Y
Shahverdyan	2020	Y	Y	N	Y	Y	Y	Y	Y	N	Y	Y	Y	Y	Y	N	Y	Y	Y
Hull	2020	Y	Y	N	P	Y	Y	Y	Y	Y	Y	Y	Y	Y	Y	N	Y	Y	Y
Beathard	2020	Y	Y	Y	P	Y	Y	Y	Y	Y	Y	Y	Y	Y	Y	N	Y	Y	Y
Mallios	2020	Y	Y	N	Y	Y	Y	Y	Y	Y	Y	Y	Y	Y	Y	Y	Y	Y	Y
Berland	2019	Y	Y	N	Y	Y	Y	Y	Y	Y	Y	Y	Y	Y	Y	N	Y	Y	Y
HeBiBi	2019	Y	Y	N	Y	Y	Y	Y	Y	Y	Y	Y	Y	N	U	Y	Y	Y	Y
Mallios	2018	Y	Y	N	Y	Y	Y	Y	Y	Y	Y	Y	Y	Y	U	U	Y	Y	Y
Hull	2018	Y	Y	Y	Y	Y	Y	Y	Y	Y	Y	Y	Y	Y	Y	U	Y	Y	Y
Lok	2017	Y	Y	Y	Y	Y	Y	Y	Y	Y	Y	Y	Y	Y	Y	Y	Y	Y	Y
Hull	2017	Y	Y	N	Y	Y	Y	Y	Y	Y	Y	Y	Y	Y	Y	N	Y	Y	Y
Rajan	2015	Y	Y	N	Y	Y	Y	Y	Y	Y	Y	Y	Y	Y	Y	U	Y	Y	Y

N, NO, not reported; P, Partially reported; U, Unclear; Y, Yes, fully reported. Items 1 to 18 indicate 18 components of quality assessment for case series.

Supplementary Table 4. Results of quality assessment of included cohort studies

Author	Year	Selection	Comparability	Outcome	Total score
Inston	2020	3	1	3	7
Shahverdyan	2021	4	1	3	8
Osofsky	2021	3	1	2	6
Harika	2021	4	2	3	9
Mordhorst	2022	3	1	3	7

Supplementary Table 5. Vascular access history of endoAVF

Author, year	Previous vascular access (n, %)	Type of vascular access		
		AVF	AVG	CVC
Mordhorst,2022	45(73.7%)	9(14.7%)	NR	36(59.0%)
Harika,2021	65(60.7%)	NR	NR	NR
Osofsky,2021	14(58.0%)	NR	NR	14(58.0%)
Shahverdyan,2021	NR	34(38.2%)	NR	48(53.9%)
Inston,2020	11(36.7%)	NR	NR	NR
Berland,2022	NR	NR	NR	NR
Kitrou,2022	21(70.0%)	NR	NR	NR
Zemela,2021	23(91.7%)	NR	NR	NR
Shahverdyan,2020	78(78.0%)	25(25.0%)	NR	53(53.0%)
Hull,2020	44(73.0%)	NR	NR	39(65.0%)
Mallios,2020	NR	NR	NR	NR
Beathard,2020	NR	NR	NR	NR
Berland,2019	31(97.0%)	0	0	31(97.0%)
HeBiBi,2019	23(67.6%)	2(5.9%)	NR	21(61.7%)
Mallios,2018	33(99.0%)	10(30.0%)	NR	23(69.0%)
Hull,2018	66(61.7%)	NR	NR	66(61.7%)
Lok,2017	61(77.0%)	22(28.0%)	NR	39(49.0%)
Hull,2017	26(100.0%)	2(8.0%)	NR	26(100.0%)

endoAVF, endovascular arteriovenous fistula; AVG, arteriovenous graft; CVC, central venous catheters; NR, not reported.
