# Supplementary figures and images for: Percutaneous endovascular arteriovenous fistula: A systematic review and meta-analysis
Source: Front Cardiovasc Med. 2022 Sep 6;9:978285. doi: 10.3389/fcvm.2022.978285 (PMC9486211; doi:10.3389/fcvm.2022.978285)

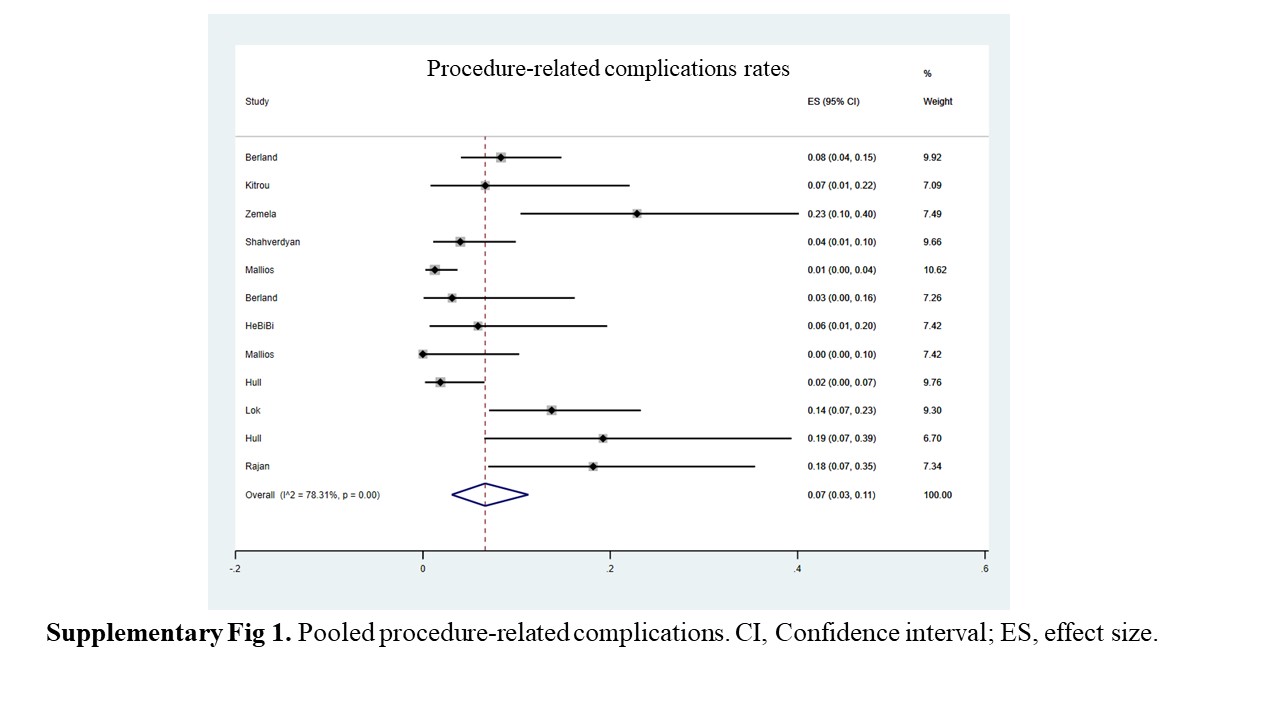

Supplement: Supplementary file 2 [file Image_1.JPEG]

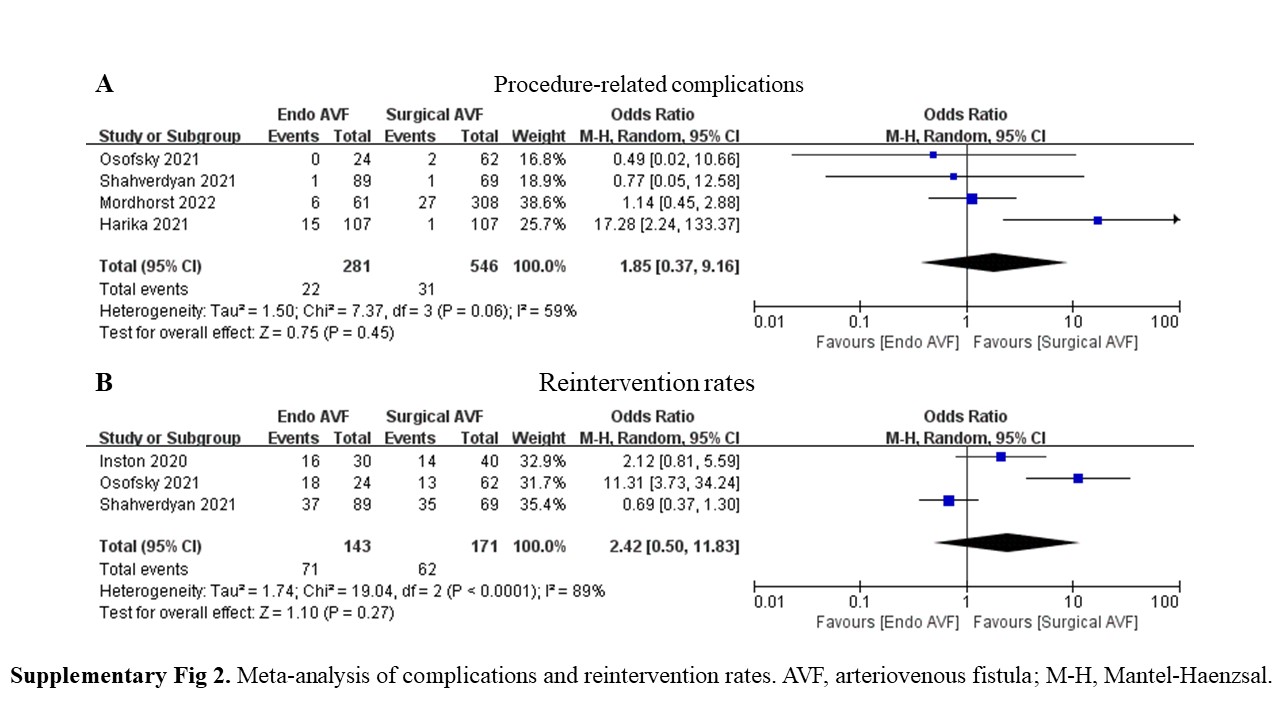

Supplement: Supplementary file 3 [file Image_2.JPEG]

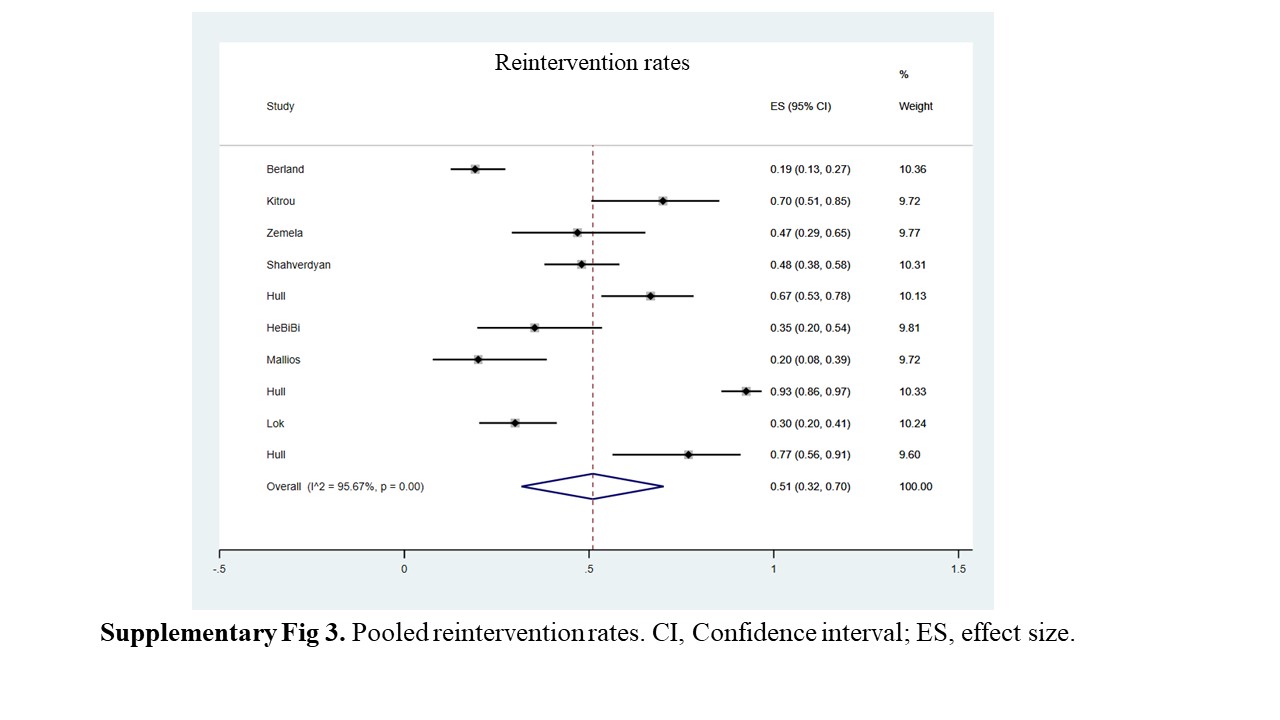

Supplement: Supplementary file 4 [file Image_3.JPEG]

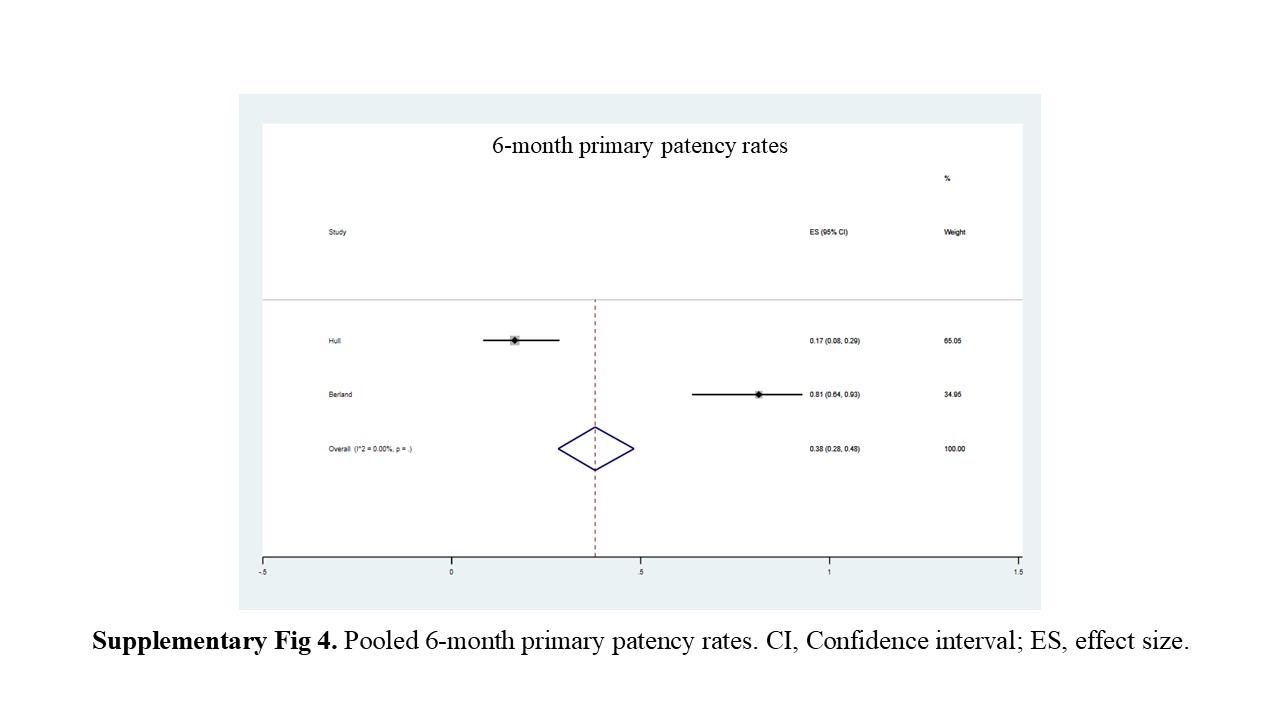

Supplement: Supplementary file 5 [file Image_4.JPEG]

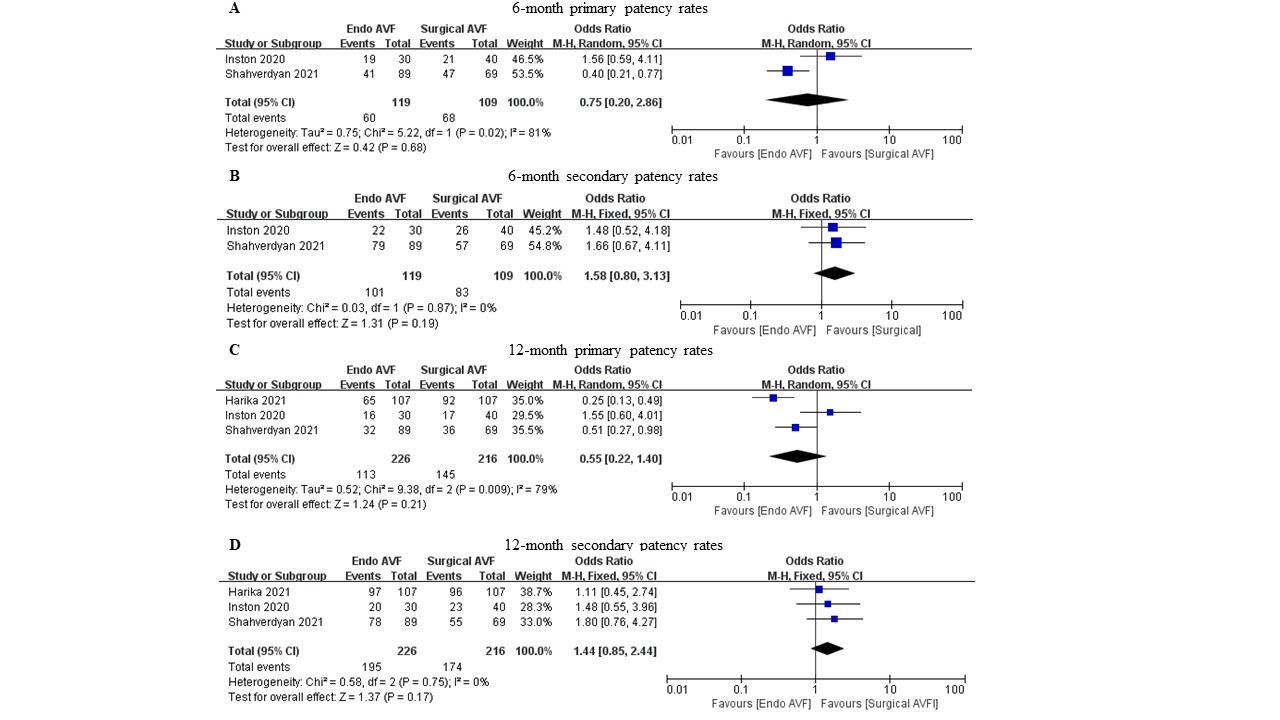

Supplement: Supplementary file 6 [file Image_5.JPEG]

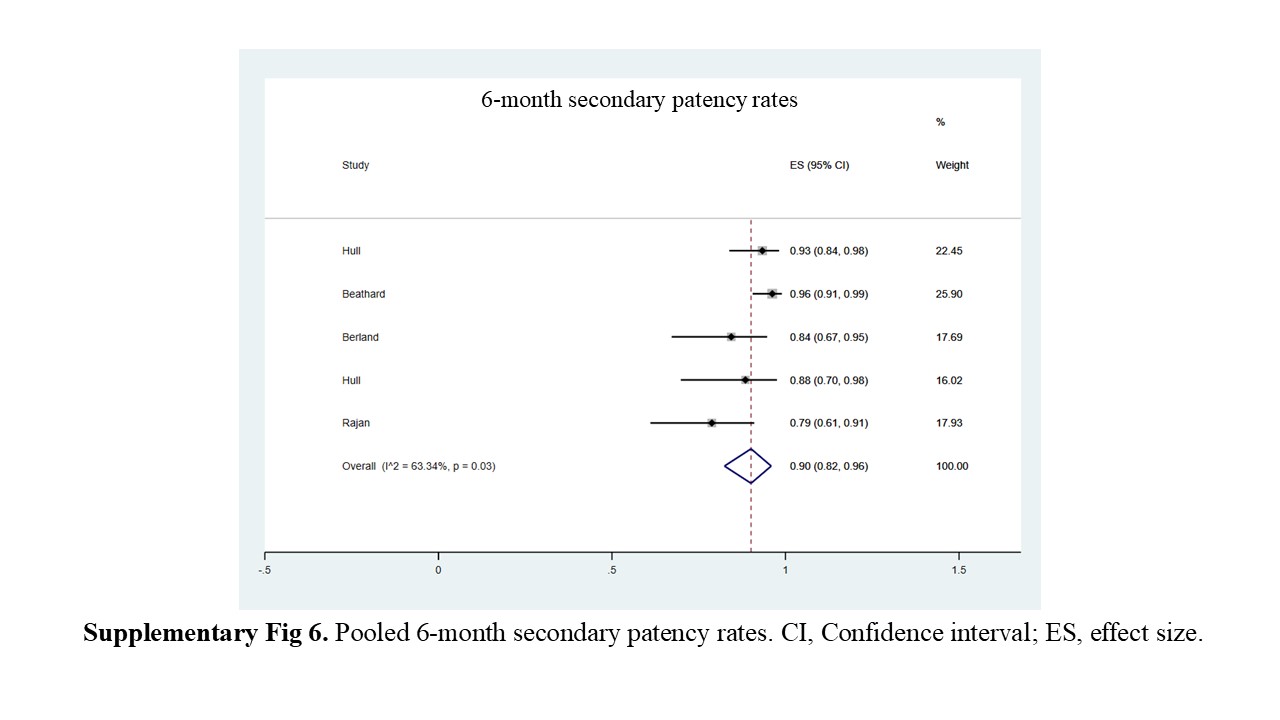

Supplement: Supplementary file 7 [file Image_6.JPEG]

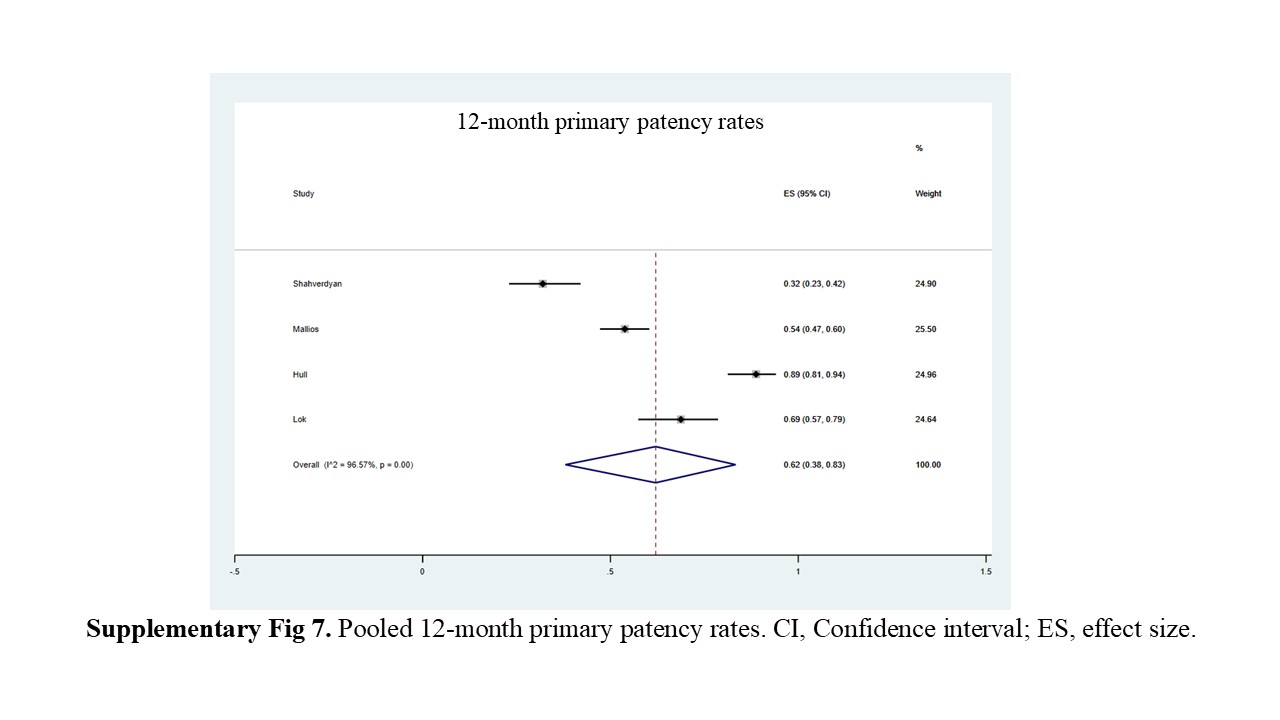

Supplement: Supplementary file 8 [file Image_7.JPEG]

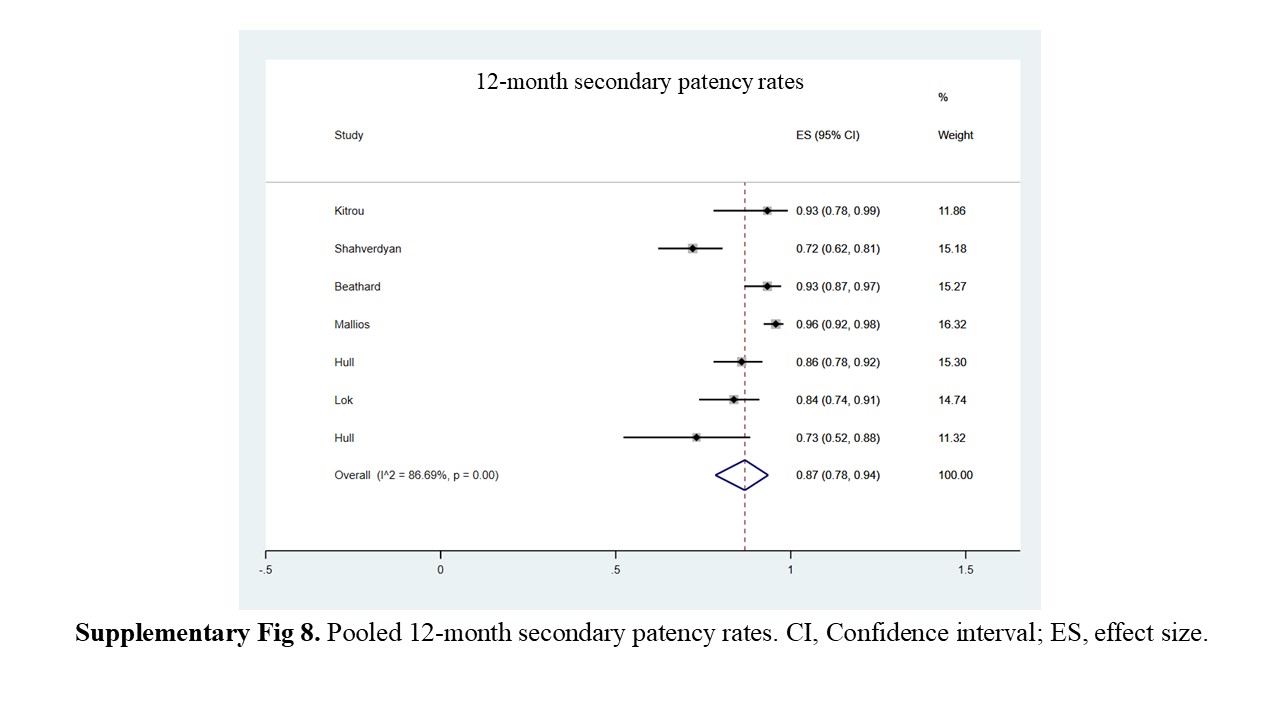

Supplement: Supplementary file 9 [file Image_8.JPEG]
